# “There is No Time” to be a Good Biocitizen: Lived Experiences of Stress and Physical Activity Among Mexican Immigrants in New York City

**DOI:** 10.1177/21582440241252236

**Published:** 2024-05-16

**Authors:** María Hernández, Alyshia Gálvez, Sandra Verdaguer, Joseph Anthony Torres-González, Kathryn P. Derose, Karen R. Flórez

**Affiliations:** 1 University of Connecticut, Storrs, CT, USA; 2 Lehman College, CUNY, Bronx, NY, USA; 3 CUNY Graduate School of Public Health and Health Policy, NY, USA; 4 Graduate Center, CUNY, NY, USA; 5 RAND Corporation, Santa Monica, CA, USA; 6 University of Massachusetts Amherst, MA, USA

**Keywords:** Mexican immigrants, acculturative stress, mental health, physical activity, biocitizen

## Abstract

This paper explores the ways in which Mexican immigrants experience, narrate, and describe stress and the extent to which it impacts their efforts at engaging in physical activity using a biocitizenship framework. Data were derived from a mixed-method study among Mexicans living in New York City recruited from a large Catholic church. The qualitative sample of 25 participated in quantitative and qualitative components of the study and as such we include some of these quantitative indicators as descriptors. Our main qualitative findings reveal that study participants experience stress and time constraint as factors that contribute to the waning of their physical and mental well-being. As such, time constraints for many of our participants were among the factors that contributed to high perceived levels of stress. They attributed this to their difficulty maintaining a physically active lifestyle due to factors like the fast-paced lifestyle in New York, working long hours, and not having enough time to exercise, though some important differences in narratives were noted across gender. Findings have implications for interventions aimed at improving the health of immigrants in general and Mexican immigrants in New York City specifically.

## Plain Language Summary

Stress is a common reaction we all experience in our daily lives, both physically and emotionally. However, when stress persists, it can have negative effects on our health. To better understand the experiences of Mexican immigrants living in NYC, we conducted individual interviews with 25 participants. We explored their thoughts on stress, physical activity, and life in NYC, as well as how the people around them influenced their efforts. The narratives shared by the participants revealed that Mexican immigrants perceive stress as having a significant impact on their physical and mental well-being. Specifically, discussions about stress affecting their health were linked to factors such as time constraints, long working hours, and not having enough time to exercise. These findings indicate that although participants desired a healthy lifestyle, they encountered barriers that hindered their ability to engage in physical activity. Additionally, our study highlighted that men and women experience stress differently. Women often felt stressed due to household responsibilities like cooking, whereas men experienced stress related to financial obligations. Despite these gender differences, both men and women described how stress negatively affected their overall health. These findings suggest that public health interventions and programs could focus on promoting physical activity to enhance the physical and mental well-being, but these would need to be tailored to fit the needs of Mexican immigrants in New York City and other similar communities.

## Introduction

In recent years, the United States has seen an increase in chronic diseases, including diabetes, heart disease, cancer, and stroke among its population, often related to poor nutrition and lack of physical activity (National Center for Chronic Disease Prevention and Health Promotion, 2022). One response of the government and public health sector to this surge in chronic disease has been to promote a “personal responsibility” approach to encouraging individuals to make healthier behavior decisions (Hook & Rose Markus, 2020). As such, this notion of living a healthy life implies that it is up to the individual to maintain a healthy lifestyle and become a “good biocitizen.”

Biocitizenship is also built around a sense of duty and membership within the nation-state (S. S. Lee, 2020). That is, biocitizenship is tied to legalistic forms of citizenship, action and effort, and social responsibility (S. S. Lee, 2020). Notably, the sociologist Halse (2009) argues that through social responsibility, a good biocitizen takes care of the nation’s welfare by helping others within their community to stay healthy. However, social responsibility puts an extra burden on women due to their role as caregivers (DiGirolamo & Salgado de Snyder, 2008). For instance, women’s responsibility is framed as raising good biocitizens by instilling healthy eating and exercise habits.

Biocitizenship may also pose significant challenges for immigrants as they in acculturate, or as they begin to espouse the norms, beliefs, and behaviors of those in the receiving context (Abraído-Lanza et al., 2016; Hovey, 2000). For example, they might feel the need to “eat a certain way, move a certain amount, and maintain a certain weight” (Greenhalgh & Carney, 2014, p. 269). However, the acculturation process is also replete with socioeconomic barriers to healthy behaviors such as economic instability, living in crowded spaces, time constraints due to work and caregiving responsibilities, and unsafe neighborhoods that preclude leisure-time physical activity (Maldonado, 2020). Thus, many immigrants find it challenging to engage in good biocitizenship through eating healthy, exercising, and even maintaining stable mental health during acculturation. This process is also often fraught with acculturative stress, or the emotional reaction to abrupt changes like the loss of a support system or language barriers (Arbona et al., 2010). While stress is a normal physical and emotional response to everyday life, prolonged stress or chronic stress may trigger negative health outcomes like metabolic syndrome, obesogenic disease, blood pressure, the deposition of fat in the abdomen, and psychiatric conditions such as depression and anxiety (Björntorp, 2001; D’Alonzo et al., 2012). Studies have linked acculturative stress specifically with mental health disorders, including depression, personality disorder, post-traumatic stress, and other mental health symptoms among immigrant groups (Bekteshi & Kang, 2020; Hovey, 2000; Hovey & Magaña, 2000).

Correlates of worse mental health outcomes among Latino immigrants include fear of deportation, marginalization, and socioeconomic disadvantages (Garcini et al., 2017). In a concept analysis, Caplan (2007) identified three major types of stressors among Latino immigrants: instrumental/environmental, social/interpersonal, and societal. Instrumental and/or environmental stressors may include lack of access to health services, unsafe neighborhoods, and poor housing (Caplan, 2007). Social and/or interpersonal stressors may be related to changes in relationships and cultural norms, like changes in gender roles. Societal stressors include interpersonal and structural discrimination and national policies that discriminate against immigrants and roll back their rights (Pierce et al., 2018). In line with this, Borges et al. (2015) found evidence that Mexican immigrants were at higher risk for onset of anxiety disorder after migration than those born in the United States. They attributed this finding to changes in family relationships, trauma experienced when crossing the border, and immigration status, among other factors (Borges et al., 2015).

Yet there is limited qualitative public health research that describes how Mexican immigrants understand their experience or narrate the concept of stress as a function of their acculturation process (Bekteshi & Kang, 2020). Furthermore, anthropological evidence suggests that health may be a crucial area of study as immigrants attempt to engage in “good biocitizenship.” That is, devoting time to developing healthy eating habits, exercising, and instilling those healthy habits in one’s family. Moreover, very little research has been conducted on how Mexican immigrants also strive for a healthy psychological and emotional well-being through their attempt to engage in “good biocitizenship.” Though public health research routinely assumes that Mexican immigrants do this well given their well-documented health advantages (Ruiz et al., 2013), anthropological research problematizes this notion and suggests that Mexican immigrants, because of their structural disadvantage and racialization in the U.S., may find ideals of biocitizenship seem to slip ever out of reach (Greenhalgh & Carney, 2014).

The objective of this paper is to further explore ideas around biocitizenship by focusing on physical activity specifically. As a health behavior, engaging in leisure-time physical activity has been associated with a wide array of physical and mental health outcomes (Arredondo et al., 2011; I.-M. Lee et al., 2012; “Surgeon General’s Report on Physical Activity and Health,” 1996).

Yet to attain the most health benefits, it is recommended that adults engage in at least 150 to 300 minutes of moderate-intensity aerobic activity, like brisk walking or fast dancing each week (U.S. Department of Health and Human Services, 2018). This is in addition to muscle-strengthening activities at least 2 days each week. Our paper further explores the extent to which Mexican immigrants can achieve such standard in the context of their everyday lives in a postmodern industrialized city like New York City, notorious for its fast-paced lifestyle. Our paper elucidates the way in which Mexican immigrants experience stress in relation to time constraints and how this affects their ability to engage in *leisure-time physical activity*. We make the distinction between leisure-time and overall physical activity since evidence suggests racial/ethnic groups have physically strenuous occupations and commutes that do substantially contribute their ability to meet these guidelines (Echeverría et al., 2019). Also, there may be an additional mental health benefit from leisure time physical activity (PA) as leisure-time PA has been shown to mediate the relationship between depressive symptoms and hypertension among Latino adults (Arredondo et al., 2011). Yet most public health research does not focus on overall physical activity, but leisure-time physical activity only.

### Study Design

This study uses a subset of qualitative data gathered for a larger mixed method study designed to understand the role of social networks among Mexicans and Mexican American in shaping diabetes and diabetes-related health behaviors like diet and physical activity. Briefly, the quantitative portion of the parent project involved participants who self-identified as Mexican, Mexican American, or Chicano (*N* = 81). All were recruited through a large Catholic Church in NYC. We used the quantitative data on personal network to calculate network-level characteristics like the density of the network, the percentage of family members in the network, and how connected the network members were to one another. We then used the network characteristics to group the potential participants into three different categories, based on how strong or weak their network connections were. These characteristics were then used to select a group of 25 participants out of the 81 for a qualitative sample. The goal of this sample was not to represent the entire population, but instead to select a small number of participants from each network connection category who could provide detailed and informative data.

### Qualitative Data Collection

Participants were contacted by phone and invited to participate in an in-depth interview, after which they would receive $25 as an incentive. If the participant was interested, they signed an informed consent form approved by the City University of New York Human Subjects Institution Review Board (IRB # 2018-1081). A total of 25 people were included in this qualitative sample for the interviews, 24 interviews were conducted in Spanish and 1 interview was conducted in English.

**Figure 1.**
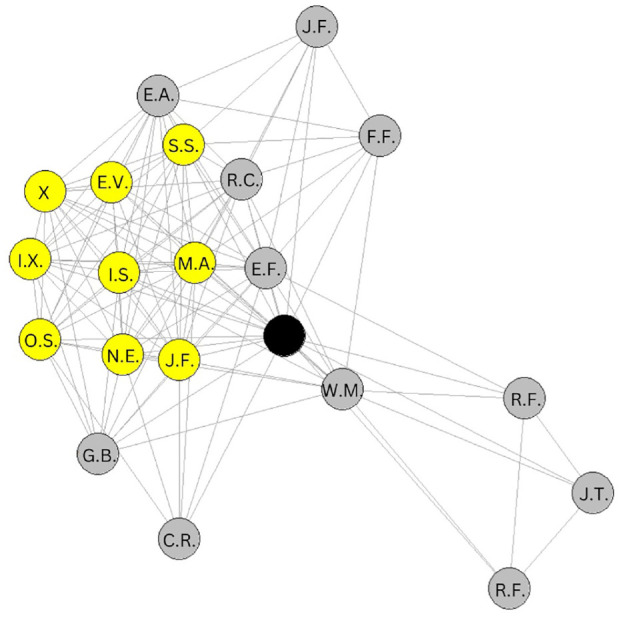
Social Network Map.

Interviews were carried out between August 2019 and February 2020. Each took between 25 and 60 minutes to complete. The interviews began with the interviewer showing a map of the participant’s social network derived from the quantitative data and explaining it (Figure 1). This tool helped the researchers to identify distinct groups of people in the participants’ social networks and to ask questions about these groups, such as how the people in the group knew each other. Interviews then followed a semi-structured guide to assist the research assistants in exploring and probing to improve the richness of the data being collected. The guide included questions to ask about distinct clusters in their network if any (e.g., “How do they know each other?”) as well as isolated alters and any alter who connected groups of alters who were highly central. This was in addition to having participants identify which alters they go to for health-related information. While the interview guide explored various health related topics, physical activity and stress were the main focus of the present manuscript.

### Coding and Data Analysis

Audio recordings were transcribed verbatim in Spanish or English. Transcripts were uploaded in their original language to Dedoose, a qualitative data analysis software program that facilitates coding and data analysis (Dedoose Version 9.0.17, 2021). A preliminary codebook was developed based on the interview guide and as themes emerged, codes were either added, deleted, or merged. Three bilingual coders coded each transcript, meeting weekly to discuss codes that need to be modified or adjusted and the categories used for this analysis are listed in Table 1. All English translations were done for this paper by the authors.

**Table 1.** Code Categories Used for Analysis.

Code	Definition
*Time*	Lack of time due to work, school, children.
*Time as an asset*	Determines how one will use one’s human capital in society and its impact on personal lives of individuals.
*Lack of time to exercise*	When they mention they have no time to exercise.
*No time for health promotion (e.g., medical checkups)*	When they mention they don’t have time for health promotion including health check-ups.
*Changes from Mexico*	When they view life in Mexico as calm rather than working all the time and having no time to see family and friends.

We also used some of the quantitative indicators available for each of the 25 participants for purely descriptive purposes. These were derived using SPSS, and include:

*Acculturative Stress Scale*: This scale was adopted by and used by the National Latino and Asian American Survey, and it assesses the stress felt in the process of adapting from the origin to the destination culture (Alegria et al., 2004). It is a nine-item scale with dichotomous responses (yes = 1 or no = 0) to questions about negative aspects of the acculturation process (e.g., feeling guilty for leaving family in a home country; being questioned about legal status). All items were summed, with higher values representing higher acculturative stress.
*Psychological Distress*: The Kessler Psychological Distress Scale (K-6) assessed participants’ distress over a period of 30 days (Kessler et al., 2003). The K-6 involves six questions about a person’s emotional state (e.g., About how often during the past 30 days did you feel nervous? Hopeless?) Participants reported how often they have experienced depressive and anxiety symptoms within the last 30 days by rating K-6 items on a Likert-type scale that range from 0 (none of the time) to 4 (all of the time). Scores of the six questions were summed to yield an overall level of psychological distress, where greater scores correspond to higher levels of psychological distress.
*Physical activity (PA)*: We used the International Physical Activity Questionnaire (IPAC) short from, which measures three types of activities: walking, moderate-intensity activities, and vigorous-intensity activities (IPAQ, version 2.0, 2004). Based on the criteria met, participants were classified in three levels of physical activity: low, moderate, and high.
*Sociodemographic characteristics*: The sociodemographic profile encompassed various factors, such as years of U.S. residency, age, gender, educational attainment, employment status, household income, marital status, general health, and health insurance coverage. Among these, years of U.S. residency and age are reported as continuous numerical variables, while the remaining factors are reported as categorical variables.

## Results

Table 2 provides demographic descriptive statistics of participants. Sixteen (64%) participants were women and 6 (34%) were men. The majority of the sample was female (*n* = 16; 64%), employed (*n* = 20; 80%), married (*n* = 16; 64%), and had health insurance (*n* = 15; 60%). Fair or poor self-rated overall health was common among the sample (*n* = 14; 56%). The mean reported age was 41.52 years (standard deviation [*SD*] = 13.04; range 20–64). Participants had lived, on average, 20.92 years in the United States (*SD* = 10.44). More than half (*n* = 15, 60%) of the sample reported having 11 years or fewer of education; however, 24% (*n* = 6) had a high school diploma or the General Educational Development, and 16% (*n* = 4) had attended at least 1 year of college or had a bachelor’s degree or higher. Most of the participants (*n* = 18, 72%) reported an annual household income <$10,000; 12% (*n* = 3) had a household income of $10,000 to $29,999 and 16% (*n* = 4) had a household income of ≥ $30,000. Slightly greater than half (*n* = 13, 52%) of participants reported high levels of physical activity, while 16% (*n* = 4) reported moderate levels of physical activity and 32% (*n* = 8) low levels of physical activity.

**Table 2.** Descriptive Characteristics of Participants (*n* = 25).

Years residency in United States, mean ±*SD*	20.92 ± 10.44
Years of age, mean ±*SD*	41.52 ± 13.04
Gender, *n* (%)	
Female, *n* (%)	16 (64%)
Male, *n* (%)	9 (36%)
Education level, *n* (%)	
<11th grade	15 (60%)
High school graduate or GED	6 (24%)
Some college and above	4 (16%)
Employed, *n* (%)	20 (80%)
Household income, *n* (%)	
<$10,000	18 (72%)
$10,000–$29,999	3 (12%)
≥$30,000	4 (16%)
Marital Status, *n* (%)	
Married or living with partner	16 (64%)
Not married and not living with partner	9 (36%)
General Health, *n* (%)	
Good, very good, or excellent	11 (44%)
Fair or poor	14 (56%)
Health insurance, *n* (%)	
Insured	15 (60%)
Uninsured	10 (40%)
K6, mean ±*SD*	4.40 ± 4.13
Acculturative Stress Scale, mean ±*SD*	3.88 ± 1.94
IPAQ, *n* (%)	
Low	8 (32%)
Moderate	4 (16%)
High	13 (52%)

**Figure 2.**
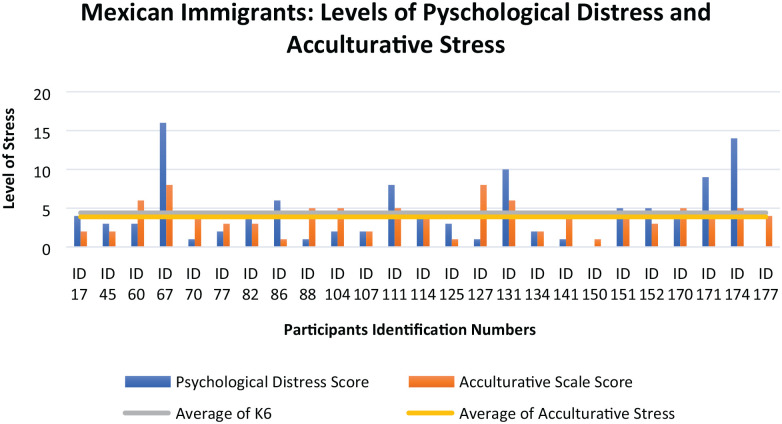
Levels of Pyschological Distress and Acculturative Stress.

The graphic below (Figure 2) represents the values of Psychological Distress (blue bar) and Acculturative Stress (orange bar) of each participant (identified by ID). The gray line and the yellow line indicate the participants average of the total score for Psychological Distress (mean = 4.40) and Acculturative Stress (mean = 3.88), respectively.

### Description of Physical Activity as Part of Their Everyday Lives

While participants in our sample did engage in some physical activity, most described a wide array of difficulties and barriers. For example, Participant [77] with low levels of physical activity expressed the need to lose weight. However, he identified both knee pain and family dynamics to be barriers to engaging in exercise.

**Participant [77]:**

Acculturative Stress Score: 3 (below the sample mean)

K6 score: 2 (below the sample mean)

*[Before] I would run a lot. I would get back from work and I would head out to run, to be able to think. But then I would take a bath, I would have dinner, and straight to bed. I am not overweight. Oh, well. Sometimes, the pressure from work and the pressure from home [is a lot]. Sometimes, it’s not even about the actual work you do, but what you failed to do or finish…*

### How Participants Describe Stress

Our participants described stress as taking a toll on their physical and mental well-being. Some articulated that stress contributes to increases in weight, depression, and anxiety. They also were aware that stress impacts their physical health due to time constraints like working long hours, having little time to prepare healthy meals, and having little time to practice self-care for a healthy lifestyle. For some scoring above the sample mean on the quantitative measure of psychological distress, they described stress as a disease or “una enfermedad.” For example, Participant [ID151], a 33-year-old woman, describes her overall health in relation to stress in the following excerpt.

**Participant [ID151]:**

Acculturative Stress Score: 4 (above the sample mean)

K6 score: 5 (above the sample mean)

*Well, right now I am sick from stress. I gained 15 pounds [inaudible 0:38:37]. Someone else told me it was normal because…to tell you that I keep biting my nails. It will be almost a month that I been like this, sleeping. I never want to eat, because I always want to sleep, and I always feel tired. Every time I want, anything that happens, something TV, it’s what I feel. I feel something uncomfortable in my chest and I cry a lot. That is because I am sick, and they sent me to the psychiatrist who will medicate me.*

Participant [ID151] described her physical and mental health as stemming from stress; she said she was sick from stress, “enferma de estres.” Being “sick from stress” has led to the deterioration of her mental health and weight gain. She also alluded to symptoms of depression, such as wanting to stay in bed and not being hungry.

While another woman, Participant [ID174], age 39, does not explicitly mention stress, she describes mental health issues she contends with, which has led her to an increase of her food consumption. Both her acculturative stress and psychological distress scores were above the mean.

**Participant [ID174]**

Acculturative Stress score: 5 (above the sample mean)

K6 score: 14 (above the sample mean)

*A couple of months ago, I began feeling a lot of anxiety and sadness. So, I am going to a clinic in where they can see me which is close to my house and…it was because I began feeling a lot of anxiety…*

*So, there was a time I would just eat…. I tried to eat normal, but suddenly I began to feel sad, really sad and I began to eat. I increased the amount of food and eating at different times. For example, at night… I was avoiding [eating large meals] because I was getting in shape.*

Participant [ID174] described feeling anxious and having a deep feeling of sadness. This feeling of sadness has not only deteriorated her mental well-being, but also led to an increase her food intake. While she is trying to get back in shape, she found it difficult to avoid eating most of the time.

### Lifestyle in NYC Compared to Mexico

Participants often described their lifestyles in NYC as being more stressful than their lives prior to migration. They recalled having more time for themselves as well as family and friends in Mexico. Several said “no hay tiempo [there is no time]” in NYC. They attributed difficulties with having enough time to interact with members of their social networks, exercise, or go to the doctor to this difference. Participant [ID127], age 50, emphasized that her sister in Mexico has more time to cook, exercise, and maintain a healthy lifestyle because life there is very different:

**Participant [ID127]:**

Acculturative Stress score: 8 (above the sample mean)

K6 Score: 1 (below the sample mean)

*Well, back there [Mexico] things are slower. I tell my sister: “You can do Zumba because back there you have more time, here we don’t have the time. Here we have to work, from work cook, after cooking and after you’re done eating you got to shower and head to bed.” We don’t do anything; however, back home life is more peaceful, and I tell her: After we are done working, we cook, and you end up eating at 7:00pm.” By around 3:00pm they eat and by the time it’s 7pm they have metabolized their food. That is why I said they have it better than us here.*

The lifestyle Participant [ID127] describes is a constant cycle of eating, sleeping, and working. The lack of time prevents her from taking care of her own body because cooking and eating takes time. For example, she felt that eating at 7:00pm did not allow for proper digestion. Because of this she felt that people living in Mexico are much healthier than those who live in the United States.

Participant [ID88] framed the stressful fast-paced lifestyle of NYC as “te está acabando”—which we translate as “is slowly killing you.” He describes the time pressure at work as making it impossible to have a peaceful life. The busyness will eventually be fatal, and the only escape from it is back in Mexico.

**Participant [ID88]**

Acculturative Stress score: 5 (above the sample mean)

K6 Score: 1 (below the sample mean)

And that is the number one disease that slowly kills you.

*[Interviewer] Stress.*

*Stress, because it slowly kills you. There is no… there is not…peaceful life that you can have. It does not exist because it always behind you, all the time, there is this rush. Including in your home, it’s like, “hey, hurry up because you will be late.” At work they tell you to hurry up because your break is almost over. Hurry up because you must finish that. Like everything is against time and everything is measured. If for some reason you lose your rhythm, you don’t finish. Or you finish late because everything is measured and that is what will finish you, this city, the stress, ¿Why do you think people take two-month vacations in their pueblos [in Mexico]? They go to places where…they can breathe fresh air, smell the grass, listen to the river, smell the wet dirt underneath the trees. What do I know, but outside the city, outside the busy rhythm.*

At age 24, Participant [ID60] had recently arrived from Mexico and he emphasized the loneliness that often coincides with relocating.

**Participant [ID60]**

Acculturative Stress score: 6 (above the sample mean)

K6 Score: 3 (below the sample mean)

*Yes, it very different. Well, I recently arrived, and I feel it different because I don’t feel the same, because back home after I was done working, I would hang out with my friends in the evening…. Not here because I don’t know many places. I can’t go out or know what to do. Plus, I don’t have anyone to go out with.*

### How Stress and Time Affect Exercise

Some participants spoke during their in-depth interviews how they would engage in physical activities by exercising 30 to 60 minutes. They mentioned dancing (e.g., Zumba), walking, soccer, and running. Men were more likely to engage in sports such as soccer and cycling, while women were more likely to engage in walking or Zumba. However, our participants also suggested that certain barriers would constrain them from exercising, including lack of time, bad weather, lack of space, work obligations, and lack of encouragement from their friends and family. Time constraints were often related to working long hours, taking public transportation, or the busy schedule of their friends and neighbors. When we asked participants with whom they exercise, they named close ties such as children, spouses, and friends. Participant [ID67], age 59, described her difficulties attending her Zumba class because of her work schedule.

**Participant [ID67]:**

Acculturative Stress score: 8 (above the sample mean)

K6 score: 16 (above the sample mean)

*We [alter in network] go twice a week. Maybe, they can go three or four times because they have Zumba every day. Because of my work, I cannot go to Zumba classes every day. Only Mondays and Fridays.*

Participant [ID134], age 53, said that a friend of hers was sometimes her impetus to take a walk in the park, but there were barriers to doing so:

**Participant [ID134]**

Acculturative Stress score: 2 (below the sample mean)

K6 score: 2 (below the sample mean)

*Sometimes I go [for walks] with [Friend’s name] who also takes her children to the park, or sometimes I go to her house. I don’t visit her often because sometimes I get a bit lazy to come back home.*

When asked whether her friend lives in Brooklyn, like Participant [ID134], she said,

*No, she lives by Pelham Bay [in the Bronx] but I must take the train and then the bus and now it’s darker much earlier.*

Participant [ID77], age 64, explained how significant it is to have a new member of his social network to exercise with. He has two sons and he said that they used to exercise with them, that they would walk, play soccer, and go to beach. But now that they have children, they do not have the time to exercise with their father. On the weekends both sons are busy with household responsibilities like laundry.

**Participant [ID77]**

Acculturative Stress score: 3 (below the sample mean)

K6 score: 2 (below the sample mean)

*Sometimes I would go with [Friend], however, she is pregnant now. We would go the gym, I would say that before I would run much more, now even going to the gym I get lazy…. Sometimes, I just paid for the membership, and I don’t go. It’s not about throwing money away; I want to go, but, what for? So, I said, “let’s go” but it’s because you…I am not sure why now, I am more the type that needs someone telling me…There needs to be someone who tells me, “Let’s go.”…. I don’t know, someone who pushes you. Or someone that wants to go with, I tell her, “Come on, let’s go” but I don’t…” But I will go to sleep.”“Let’s go,”“No, you go.”*

Participant [ID141], age 42, described the city environment as not conducive to exercise. When asked if she exercised, she said that she didn’t and that the lifestyle in Mexico is different: “You walk a lot, you exercise much more.”

**Participant [ID141]**

Acculturative Stress score: 4 (above the sample mean)

K6 score: 1(below the sample mean)

*No because in Mexico, like there was like enough space to run, jump, and things like that. Right here you really can’t.*

### Gender Difference

While most of our participants experienced stress, it was portrayed differently by female and male participants. Women were more likely than men to reference their household responsibilities as stressful such as cooking, working outside the household, and encouraging their family members to be healthy. Even when they took time for physical activity, they framed it as something they undertook for others. Participant [ID111] said that walking around the park was something her kids enjoyed as well as her husband, and therefore she did it with them.

Participant [ID150], age 43, attributed her stress to her responsibility to care for her children.

**Participant [ID150]**

Acculturative stress score: 1 (below the sample mean)

K6 score: 0 (below the sample mean)

*This week I went to find out how much we had to pay for the bills, so I am analyzing, if I work with my husband, I leave the house at 4am or 5am. […] I have a daughter who is in high school, she is 13 years old, so if my daughter, I take my daughter to school at 7am, so I tell my husband, “You’re going to take her.”“I will not take her to school,” he told me, “I have to sleep” because he goes at 10 am to work. […] Sometimes he has 3 or 4 other days or 3 days [at work]. But why doesn’t he help out? But since I am always the one taking our daughter to school, he is accustomed to that, and so he does not take her to school.*

Men’s stressors were likewise gendered, relating generally to working long hours and financial obligations. Participant [ID170] was concerned about the stress on her husband.

**Participant [ID170]**

Acculturative Stress score: 5 (above the sample mean)

K6 score: 4 (above the sample mean)

*Yes, he is always working. […] Yes, he leaves at 7 and is not back until 11-11:30 because he does not want me to work.*

Participant [ID177] describes the toll of his job managing a community soccer team to supplement his income:

**Participant [ID177]**

Acculturative Stress score: 4 (above the sample mean)

K6 score: 0 (below the sample mean)

*They are making me do the schedules and the teams are calling me and they are scheduling tables. I have a lot on my plate, I don’t have a lot of time, so right now I get home, right now I have to make copies, make copies and send them to the teams because we have various fields, so I have to do it, I have to make the schedules, I have to make the schedule tables sometimes every day, so I just get home and sit down [to do it]. I go to sleep at 12am, 12:30am, sometimes at 1am, and I have to get up by 5am, every day, except for Fridays, because at games at a gym and Saturdays too.*

This sleep schedule is clearly harmful, but Participant [ID177]’s financial worries compelled him to work extremely long hours.

## Discussion

Findings reveal that Mexican immigrants experience stress and time constraints as factors that contribute to the waning of their physical and mental well-being. Our participants’ lived experiences of stress and time constraints were often related to the fast-paced lifestyle, working long hours, and family obligations. This often led to not having enough time to exercise and was portrayed as yet another factor preventing them from maintaining a more healthful lifestyle and engaging in “good” biocitizenship related to their level of physical activity. As Greenhalgh (2012) argued, a good biocitizen’s duty is the maintenance and surveillance of one’s body, including maintaining a normal BMI. Thus, failing to maintain a BMI within a specific range not only positions Mexican immigrants as “overweight” but as “bad citizens.” Ideals of “good biocitizenship” remain ever out of reach for racialized and classed people who are seen to fall outside of the norms. In this way, not only might acculturative stress have to do with pressure to subscribe to ideals of ideological, linguistic, and economic achievement, but even body shape and weight. Most participants in this study did not meet the physical activity guidelines on both aerobic and anaerobic exercise (Elgaddal et al., 2022), despite the robust evidence that engaging in physical activity helps with weight and stress management and that occupational and transportation related physical activity matters for racial/ethnic groups (Echeverría et al., 2019; Ottenbacher et al., 2014; Rohrer et al., 2005). Indeed, participants described stress as a “disease” or “una enfermedad” that led to other health conditions such as depression and weight gain. Lack of energy and willpower were described as disease-like symptoms that laced the narratives of other low-income Latina participants in similar qualitative inquiries (Chang et al., 2018).

Other participants described stress as being on the “go” or “estar corriendo.” By this they meant catching the subways on time, running errands quickly like food shopping, and cooking a quick meal. This feeling of time scarcity or people’s perceptions or feeling of not having enough time is generally common in highly industrialized cities like New York City (Jabs & Devine, 2006). Interventions to improve physical activity among Latinos show promising short as well as long term results (Marcus et al., 2021, 2022) though these have not been rolled out in NYC. Reflecting on the narratives of our participants highlights the need that at least in the NYC context, immigrant Latinos may benefit from PA interventions that use a whole-health approach by linking the physical activity with improved psychological outcomes. We might also interrogate norms of physical activity that prize certain kinds of exertion as “exercise” and others as “work,” valorizing those available to people who have time outside of the workday for exercise as a form of leisure, and failing to credit those that are part of one’s workday, although there may be distinct health benefit in particular mental health (Arredondo et al., 2011). Moreover, our participants constantly compared their stress experience in the US and Mexico, which highlights the importance of acculturative stress as well. Prior to migration, life in Mexico was more peaceful and they had more time to engage in other activities including exercising and spending time with friends and family. Some participants emphasized that their families back in Mexico have better health than they do because they have more time to take care of their health. In other words, Mexican immigrants know about the importance of eating healthy and exercising but also recognize the abrupt lifestyle changes during migration, including difficulties eating healthfully and engaging in physical activity and changes in their mental health. Although they tried to integrate heathy practices, they face barriers to doing so (Greenhalgh & Carney, 2014). In this way, we can see that the ways that immigrants narrate what they describe to be their healthy lifestyles prior to migration conflict with new narratives of biocitizenship after migration that frame certain body silhouettes as an ideal and rounder silhouette as a moral failure, even while the overall exigencies on their time and energy are seen by many immigrants to be stressful and unhealthy (Halse, 2009).

Our findings also suggest that stress and time constraint were expressed differently by male and female participants. For women, stress was associated with the burden of their “second shift” of unpaid domestic labor. Our women participants were often the ones in charge of childcare, grocery shopping, cleaning, and other household responsibilities, and encouraging their partners to seek medical care, while also keeping up with their own jobs. This aligns with findings by Rodriguez et al. (2016), whose qualitative study found that women held more responsibility at home than their spouses even when they both worked full-time. Past research has documented the resulting stress on women and its negative effect on their health (Popovic-Lipovac & Strasser, 2015; Rodriguez et al., 2016; Williams et al, 1991). Women participants also experience double marginalization, as women and immigrants (Popovic-Lipovac & Strasser, 2015). Evidence suggests Mexican women are socialized to be the primary caretakers for their families’ even in the face of their own individual needs (DiGirolamo & Salgado de Snyder, 2008; McDermott & Mendez-Luck, 2018). Yet Mexican immigrant women may subscribe to gender roles that primarily prize their commitment to and care for their family (Gutmann,1997; Hubbell, 1993; Jezzini et al., 2008). That is, they prioritize their families above their own needs and frame activities such as exercising as “leisure” or something that can be done only when other responsibilities have been met. That is, they saw physical activity as a selfish indulgence rather than a health-promoting behavior (D’Alonzo, 2012). Our finding that some women were more likely to exercise when they could combine it with caregiving aligns with this concept. Women saw promoting exercise as something healthy for all members of the family to do together and to spend time together. In this way, some women maximized their perceived duty to encourage their family members to engage in physical activity as an opportunity to engage in exercise themselves. If their own physical activity was viewed as being in competition with caregiving, many women described not being able or willing to prioritize it. More often, it seemed, domestic responsibilities like childcare constrained women’s abilities to engage in physical activity. As such Greenhalgh and Carney (2014) highlight that the burden of biocitizenship pushes mothers to become responsible for the health of their family including maintaining their children “fit.” As such, a “good” biocitizen not only cares for herself but also cares for others (Greenhalgh, 2015, p. 23). This is also in line with public health studies that show differences in amount of PA by gender, with lower levels of PA reported among Latinas (Abraído-Lanza, et al., 2017; Arredondo et al., 2016; Casper et al., 2013; Congello and Koniak-Griffin, 2018; Martin et al., 2017).

For the male participants, the cultural construct of familism helps frame their narratives since it focuses on the role and obligation of family members in Latino families (Mendez-Luck & Anthony, 2016). Specifically, men emphasized their obligation to support their families economically as constraining their time to take care of their health. These masculine roles are often interrelated with being the family protectors and provider, which encourages men with the motivation to work and at the same time to ignore their health problems (Daniel-Ulloa et al., 2018). More than women, men said that they work long hours and have little time for medical appointments. This finding is congruent with a qualitative finding by Daniel-Ulloa et al. (2018) that found stressors for Latino men stem from the need to work, the anxiety of getting fired, and not been able to support their families financially. These stressors in turn, pushed men to not prioritize their own health. Future research should explore whether mindful physical practices such as yoga, would be culturally acceptable to Latino men working in physically demanding occupations since this practice has been shown to reduce stress levels and relieve physical pain (e.g., neck pain, tension headaches) (Li et al., 2019; Vollbehr et al., 2018; Wang & Szabo, 2020).

## Limitations

Our study has some limitations that should be kept in mind when interpreting our study results. Firstly, during the interviews, we did not engage our participants in conversations around the concept of stress, time constraint, and gender dynamics. Instead, we asked our participants to describe how their social network impacts their overall health, including eating habits and physical activities. Discussion of time and stress emerged organically as a theme in interviews. Secondly, our findings on the narratives of stress and time constraint by our participants cannot be generalized to all Mexican immigrants; we drew on a small sample of low-income Mexicans immigrants living in the Bronx, NY. Thirdly, most participants in our study were women. As well, men generally had less than women to say regarding health-related topics on, such as exercising, eating habits, and meal preparation. Lastly, our study did not seek to explore gender differences. This limited the depth of our analysis on how Mexican men and women may experience and narrate stress and time constraints differently. Hence, future research is needed to grasp such gender differences and how they affect stress and time constraints. Nonetheless, our study provides insight into how acculturative stress affects Mexicans’ health through different social factors including how they perceived that stress impacts their health.

## Conclusions

Our research highlights that Mexican immigrants do indeed strive to be “good biocitizens,” however, they encounter various factors that impact their overall health. For one, acculturative stress and gender contributed to the complexity and difficulties in engaging in physical activity, particularly leisure-time physical activity. Secondly, we also found that while the idea of biocitizenship among the participants centered on physical health, participants also considered the impact of mental well-being on overall health to be an important component of being a good biocitizen. This study aimed to contribute to the existing scholarly knowledge about Mexican and Mexican American immigrants’ insight and narrative of stress and time constraints and how these affected their ability to engage in physical activity. Our findings have implications for the dwindling health advantages documented in this group, as well as intervention work attempting to increase this health behavior in this vulnerable population. These interventions need to take an approach that does not blame individuals for their lack of trying but instead addresses the structures that make it difficult for this population to fulfill their roles as good biocitizens.
